# Isolation Rate and Antimicrobial Susceptibility Profile of Enterobacteriaceae Isolated from Wastewater of Jimma Medical Center, Jimma, Southwest Ethiopia

**DOI:** 10.4314/ejhs.v35i4.7

**Published:** 2025-07

**Authors:** Hasen Husein, Seid Tiku Mereta, Mekidim Mekonnen, Getenet Beyene

**Affiliations:** 1 Department of Medical Laboratory Sciences, School of Medicine, Madda Walabu University, Bale, Ethiopia; 2 Department of Environmental Health Sciences and Technology, Institute of Health, Jimma University, Jimma, Ethiopia; 3 Department of Medical Laboratory Sciences, Institute of Health, Jimma University, Jimma, Ethiopia

**Keywords:** Antimicrobial Susceptibility, Enterobacteriaceae, Hospital Wastewater, Jimma Medical Center

## Abstract

**Background:**

In many developing countries, including Ethiopia, most hospitals and healthcare facilities lack proper wastewater treatment systems. As a result, untreated wastewater is directly discharged directly into nearby water bodies, posing significant environmental and public health risks. This study aimed to determine the isolation rate and antimicrobial susceptibility profile of Enterobacteriaceae species found in wastewater from Jimma Medical Center (JMC).

**Methods:**

A cross-sectional study was conducted between July and August 2022 on 60 hospital wastewater samples collected from ten different sites within JMC. Enterobacteriaceae were isolated and identified using standard bacteriological techniques. Antimicrobial susceptibility testing was performed using the Kirby–Bauer disk diffusion method. Data analysis was conducted using SPSS version 25.0 and Microsoft Excel.

**Results:**

A total of seven bacterial genera were identified. Escherichia coli (31 isolates, 51.7%) and Klebsiella spp. (27 isolates, 45.0%) were the most frequently isolated species. The isolates exhibited high resistance rates to Amoxicillin-Clavulanic acid (88.4%), Ampicillin (87.0%), Tetracycline (76.5%), and Trimethoprim-Sulfamethoxazole (71.9%). Notably, 101 out of 114 isolates (88.6%) were multidrug-resistant (MDR).

**Conclusion:**

The high prevalence of multidrug-resistant Enterobacteriaceae in hospital wastewater indicates a serious public health concern, as these bacteria may serve as reservoirs of resistance genes that can be transferred to other pathogenic strains. This highlights the urgent need for the implementation of effective wastewater treatment systems at healthcare facilities.

## Introduction

Hospital wastewater (HWW) poses a major risk in developing countries, where most healthcare wastewater is poorly managed and discharged into surface waters, which can leach into surrounding groundwater (1). Hospital wastewater is considered as one of the major reservoirs of pathogenic bacteria. The most common pathogenic Enterobacteriaceae species found in hospital wastewater include *Salmonella* spp., *Shigella* spp., *Escherichia coli* (E. coli), *Klebsiella* spp., *Yersinia* spp., *Serratia* spp., and *Enterobacter* spp. (2, 3).

Hospital wastewater contains large quantities of antibiotic-resistant bacteria and residual antibiotic concentrations that can inhibit the growth of susceptible bacteria, thereby increasing the prevalence of resistant strains in the receiving water bodies (4, 5). In least developed countries such as Ethiopia, wastewater from most hospitals is released directly into nearby rivers and streams without any form of treatment (3, 6). These polluted rivers are often used for irrigation by small-scale farmers who grow vegetables, creating a potential route for transmission of resistant pathogenic bacteria to both farmers and consumers (7).

This situation indicates that wastewater discharged from municipal sewage treatment plants or hospital wastewater treatment plants (WWTPs) can be a significant source of environmental antibiotic-resistant bacteria (ARB). Assessing the types of pathogens present in hospital wastewater, along with their antimicrobial susceptibility patterns, provides important insight into the potential risk of antimicrobial-resistant bacteria spreading to downstream communities, resulting in serious public health consequences.

To our knowledge, limited data is available on the prevalence and antimicrobial susceptibility profiles of bacterial pathogens isolated from hospital wastewater in Ethiopia, particularly in the current study area, Jimma Medical Center. Therefore, the purpose of this study was to determine the isolation rate and antimicrobial susceptibility profile of Enterobacteriaceae isolated from hospital wastewater at Jimma Medical Center, Jimma Town, Southwestern Ethiopia.

## Materials and Methods

**Study area and period**: This study was conducted at Jimma Medical Center (JMC), located in Jimma Town, Oromia Region, approximately 350 km southwest of Addis Ababa, Ethiopia. The hospital discharges its liquid waste directly into drainage ditches, which eventually flow into the surrounding environment without any form of treatment, leading to significant contamination of nearby rivers and streams. The study was carried out from July 1, 2022, to August 30, 2022.

**Sampling technique**: A total of sixty wastewater samples were collected from ten sampling locations (Table 1). Thirty samples were collected in the morning (10:00–10:30 AM), and the remaining thirty samples were collected in the afternoon (2:00–2:30 PM).

**Table 1:** Occurrences of Enterobacteriaceae isolates against sampling site and time at JMC, Jimma, and Southwest Ethiopia, July to August 2022

Variables		Total samples N (%)	Sample Positive N (%)	Sample Negative N (%)	Enterobacteriaceae isolate recovered N (%)
Site1		6(10)	5(9.1)	1(20)	9(7.9)
Site2		6(10)	5(9.1)	1(20)	5(4.4)
Site3		6(10)	6(10.9)	0	12(10.5)
Site4		6(10)	6(10.9)	0	7(6.1)
Mixed1		6(10)	5(9.1)	1(20)	7(6.1)
Mixed2		6(10)	6(10.9)	0	22(19.3)
Mixed3		6(10)	5(9.1)	1(20)	10(8.8)
Mixed4		6(10)	5(9.1)	1(20)	10(8.8)
Mixed5		6(10)	6(10.9)	0	10(8.8)
Point1		6(10)	6(10.9)	0	22(19.3)
Total		60	55 (91.7)	5(8.3)	114(100)
Time	Morning	30(50)	25(41.7)	5(8.3)	66(57.9)
	Afternoon	30(50)	30(30)	0	48(42.1)
Total		60(100)	55(91.7%)	5(8.3)	114(100)

*Note: Note: Site1 = Psychiatric unit, Site2 = Ophthalmology unit, Site3= Antiretroviral therapy (ART) collection tanker, Site4 = Biomedical center unit, Mixed1=Outpatient department (OPD) ward, Pharmacy unit and Administrative office, Mixed2 = Laboratory unit, Radiology unit and Obstetrics and Gynecology ward, Mixed3= Kitchen room/Cafeteria, Laundry room and Surgical ward A, Mixed4= Medical and Pediatric ward, Mixed5 = Covid19 center, Surgical unit B and Dental clinic unit and Point1 = waste water from all source

**Sample collection, isolation, and identification of Enterobacteriaceae**: From each site, 100 mL of wastewater sample was collected using clean and sterile 150 mL microbiological glass bottles (8). The samples were transported to the Microbiology Laboratory of the School of Laboratory Sciences for culturing, isolation, identification, and antimicrobial susceptibility testing. If any delay occurred, the samples were stored at 4°C.

Bacterial isolation was performed by mixing 1 mL of the wastewater sample with 9 mL of normal saline and serially diluting it (10^−1^ to 10^−^^5^). From each dilution, 0.1 mL was inoculated onto agar plates. Plates with 30–300 colony-forming units (CFU) per mL were considered positive and selected for further analysis according to standard laboratory procedures (9).

A 0.1 mL aliquot of the serially diluted sample was inoculated using a sterile inoculating loop onto MacConkey agar (Oxoid, Hampshire, UK). Lactose-fermenting colonies (pink-colored) were subcultured on Eosin Methylene Blue (EMB) agar (Merck, Darmstadt, Germany). To enrich *Salmonella* and *Shigella* species, Selenite F Broth was used, and the cultures were subsequently subcultured on Xylose Lysine Deoxycholate (XLD) agar. All plates were incubated aerobically at 37°C for 24 hours. Isolated bacteria, both lactose fermenters and non-lactose fermenters, were further identified using a battery of biochemical tests based on standard laboratory methods (10, 11).

**Antimicrobial susceptibility testing**: Antimicrobial susceptibility testing was conducted using the Kirby–Bauer disc diffusion method, following the guidelines of the Clinical and Laboratory Standards Institute (CLSI) (12). The antibiotic discs used included: Ampicillin (10 µg), Amoxicillin/clavulanic acid (20/10 µg), Ceftriaxone (30 µg), Ciprofloxacin (5 µg), Tetracycline (30 µg), Gentamicin (10 µg), Trimethoprim/sulfamethoxazole (1.25/23.75 µg), Chloramphenicol (30 µg), and Meropenem (10 µg). Quality control was maintained using *Escherichia coli* (ATCC 25922) and *Klebsiella pneumoniae* (ATCC 700603) strains. All antibiotic discs were obtained from Oxoid Limited, Basingstoke, Hampshire, UK.

**Data analysis**: Data were edited, cleaned, entered, and analyzed using the Statistical Package for Social Sciences (SPSS) version 25.0 (SPSS Inc., Chicago, USA) and Microsoft Excel. Descriptive statistics, including frequencies and percentages, were used. The results are presented in text, tables, and graphs.

Ethics: Ethical approval was granted from the Institutional Review Board of Institute of Health, Jimma University (IHRPG 181/2022).

## Results

**Occurrence of Enterobacteriaceae isolates by sampling unit of JMC**: A total of 60 hospital wastewater samples were collected and analyzed for the presence of Enterobacteriaceae. Out of these, 55 samples (91.7%) were positive for one or more isolates. A total of 114 Enterobacteriaceae isolates were recovered.

The highest number of isolates (22; 19.3%) were obtained from Point 1 (wastewater from all sources) and Mixed Site 2 (a combination of the Laboratory Unit, Radiology Unit, and Obstetrics and Gynecology Ward). The fewest isolates were recovered from Site 2 (Ophthalmology Unit), which yielded 5 isolates (4.4%). All samples collected in the morning were positive for one or more bacterial species (Table 1).

**Figure 1:**
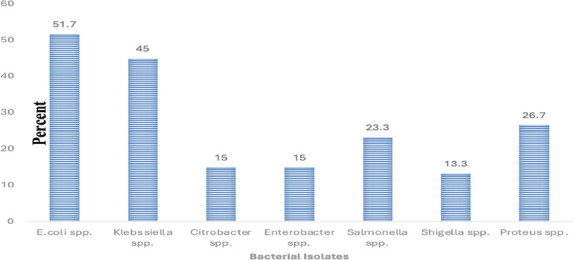
Frequency of Enterobacteriaceae isolated from JMC wastewater Jimma, Southwest Ethiopia from July to August 2022

**Prevalence of Enterobacteriaceae Isolate**: - A total of 114 bacterial isolates of Enterobacteriacae were recovered which belong to seven genera namely:- Escherchia, Klebssiella, Citrobacter, Enterobacter, Salmonella, Shigella and Proteus. Out of 114 isolates,31(51.7%), 27 (45.0%), 9(15%), 9(15%),14(23.3%), 8(13.3%) and 16(26.7%) samples were positive for *E.coli, Klebssiella* spp., *Citrobacter* spp., *Enterobacter* spp., *Salmonella* spp., *Shigella* spp., and *Proteus* spp. respectively (Figure 1).

**Drug susceptibility profile of Enterobacteriaceae**: The isolated bacteria were tested against nine different antibacterial agents. Among the isolates, *E. coli, Salmonella* spp., *Shigella* spp., and *Proteus* spp. showed high levels of resistance to Ampicillin, with resistance rates of 87.1%, 92.3%, 87.5%, and 81.3%, respectively. These isolates also exhibited high resistance to Amoxicillin-Clavulanic acid, with corresponding resistance rates of 90.3%, 85.7%, 87.5%, and 87.5%.

The majority of *Klebsiella* spp. (81.5%) and *E. coli* (83.8%) were resistant to Tetracycline. High levels of resistance to Trimethoprim/Sulfamethoxazole were observed among *Klebsiella* spp. (96.3%), *Salmonella* spp. (78.6%), *Proteus* spp. (81.3%), and *Shigella* spp. (75.0%). Resistance to Ciprofloxacin was also considerable, with 87.5% of *Shigella* spp., 81.5% of *Klebsiella* spp., 77.8% of *Citrobacter* spp., and 74.2% of *E. coli* isolates showing resistance.

High resistance to Ceftriaxone was recorded among *Shigella* spp. (75.0%), *Proteus* spp. (68.8%), *Citrobacter* spp. (66.7%), and *E. coli* (64.5%). On the other hand, the highest sensitivity rates among all isolates were observed against Meropenem (ranging from 77.8% to 100%) and Chloramphenicol (ranging from 71.0% to 88.9%). All (100%) *Shigella* spp. isolates were sensitive to Meropenem, and all (100%) *E. coli* isolates were sensitive to Chloramphenicol. Additionally, Gentamicin showed high effectiveness, with sensitivity rates of 93.4% in Proteus spp., 87.5% in *Shigella* spp., 85.7% in *Salmonella* spp., and 83.9% in *E. coli* (Table 2).

**Table 2:** Antibiotic susceptibility profile Enterobactriaceae isolates from Wastewater of JMC, Jimma, Southwest Ethiopia, July to August 2022 (n= 114)

Bacterial isolate(n)	Susceptibility	Antibiotics Susceptibility profile N (%)								

		Amp	AML	CRO	CIP	Gen	STX	TE	C	MEM
*E.coli* (n=31)	R	27(87.1)	28(90.3)	20(64.5)	23(74.2)	5(16.1)	15(48.4)	26(83.9)	9(29.0)	2(6.5)
	S	4(12.9)	3(9.7)	11(35.5)	8(25.8)	26(83.9)	16(51.6)	5(16.1)	22(71.0)	29(93.5)
*Klebssiella* spp.(n=27)	R	NT	NT	17(62.9)	22(81.5)	13(48.1)	26(96.3)	22(81.5)	5(18.5)	2(7.4)
	S	NT	NT	10(37)	5(7.4)	14(51.9)	1(3.7)	5(18.5)	22(81.5)	25(92.6)
*Citrobacter* spp.(n=9)	R	NT	NT	6(66.7)	7(77.8)	3(33.3)	4(44.4)	6(66.7)	2(22.2)	2(22.2)
	S	NT	NT	3(33.3)	2(22.2)	6(66.7)	5(55.6)	3(33.3)	7(77.8)	7(77.8)
*Enterobacter* spp.(n=9)	R	NT	NT	5(55.6)	3(33.3)	4(44.4)	7(77.8)	8(88.9)	1(11.1)	2(22.2)
	S	NT	NT	4(44.4)	6(66.7)	5(55.6)	2(22.2)	1(11.1)	8(88.9)	7(77.8)
*Salmonella* spp. (n=14)	R	13(92.3)	12(85.7)	9(64.3)	8(57.1)	2(14.3)	11(78.6)	7(50)	2(14.3)	1(7.1)
	S	1(7.7)	2(14.3)	5(35.7)	6(42.8)	12(85.7)	3(21.4)	7(50)	12(85.7)	13(93)
*Shigella* spp. (n=8)	R	7(87.5)	7(87.5)	6(75.0)	7(87.5)	1(12.5)	6(75.0)	6(75.0)	2(25)	0
	S	1(12.5)	1(12.5)	2(25.0)	1(12.5)	7(87.5	2(25.0)	2(25.0)	6(75)	8(100)
*Proteus* spp.(n=16)	R	13(81.3)	14(87.5)	11(78.6)	8(50.0)	1(37.5)	13(81.3)	NT	2(12.5)	2(12.5)
	S	3(18.8)	2(12.5)	5(31.3)	8(50)	15(93.4)	3(18.8)	NT	14(87.5)	14(87.5)
All bacteria (n=114)	R	60(87.0)	61(88.4)	74(65.0)	78(68.4)	29(24.4)	82(71.9)	75(76.5)	23(20.2)	11(9.6)
	S	9(13.0)	8(11.6)	40(35.0)	36(31.6)	85(74.6)	32(28.1)	23(23.5)	91(79.8)	103(90.4)

*Note: R=Resistance, S= Susceptible, Amp=Ampicillin AML = Amoxicillin-Clavulinic acid, CRO = Ceftriaxone, CIP=Ciprofloxacin, Gen = Gentamicin, SXT =Trimethoprim- sulfamethoxazole, TE = Tetracyclines, C=Chloramphenicol, MEM = Meropenem, NT = Not tested

**Multidrug resistance patterns of the bacterial isolates**: The overall MDR (resistance to at least 3 or more classes of antimicrobial agents) rate among the identified isolate was 101/114 (88.6%). Among the MDR isolates, about 46/114(40.2%) were resistant to five and more antibiotics. High MDR percentages were observed among *Shigella* spp. (100%), *E. coli* (93.5%), *Salmonella* spp. (92.8%), *Klebsiella* spp. (88.9%) and *Proteus* spp. (87.5%) (Table 3).

**Table 3:** Multi drug resistance pattern of Enterobactriaceae isolates from Wastewater of JMC, Jimma, Southwest Ethiopia, July to August 2022

Isolates	Multi drug resistance profile, N (%)						

	R0	R1	R2	R3	R4	≥R5	MDR
*E. coli* (n= 31)	0	0	2(22.2)	6(18.8)	7(30.4)	16(34.8)	29(93.5)
*Klebssiella* spp.(n= 27)	0	1(25)	2(22.2)	13(40.6)	4(17.4)	7(15.2)	24(88.9)
*Citrobacter* spp.(n= 9)	0	1(25)	2(22.2)	2(6.3)	2(8.7)	2(4.3)	6(66.7)
*Enterobacter* spp. (n= 9)	0	1(25)	1(11.1)	3(9.4)	1(4.4)	3(6.5)	7(77.8)
*Salmonella* spp. (n=14)	0	1(25)	0	3(9.4)	2(8.7)	8(17.4)	13(92.8)
*Shigella* spp. (n= 8)	0	0	0	1(3.1)	2(8.7)	5(10.9)	8(100)
*Proteus* spp. (n= 16)	0	0	2(22.2)	4(12.5)	5(21.7)	5(10.9)	14(87.5)
Total n= 114	0	4(3.5)	9(8.0)	32(28.1)	23(20.2)	46(40.2)	101(88.6)

*Note: R0= Susceptible to all antibiotics tested, R1= Resistance to one class of antimicrobial, R2=Resistance to two class of antimicrobial, R3= Resistance to three class of antimicrobial, R4= Resistance to four class of antimicrobial, ≥R5=Resistance to five or more class of antimicrobial, MDR= Multi-drug resistant

## Discussion

The positivity patterns of all samples collected from various sampling sites were largely consistent, with all morning samples testing positive. This may be attributed to the possibility that bacteria multiply overnight in patients' bodies and are excreted in higher concentrations in the morning.

The most frequently isolated organisms in this study were *E. coli* (31; 51.7%), *Klebsiella* spp. (27; 45.0%), *Proteus* spp. (16; 26.7%), and *Salmonella* spp. (14; 23.3%). The predominance of *E. coli* and *K. pneumoniae* is comparable with findings from other countries. For instance, a study conducted in China reported *E. coli* and *K. pneumoniae* at rates of 56.5% and 27.4%, respectively (13); in Bangladesh, both *E. coli* and *Klebsiella* spp. were reported at 30.7% (14); and in Kenya, the isolation rates for *E. coli* and *Klebsiella* spp. were 43.2% and 23.2%, respectively (15). Similar results were observed in Ethiopia: *E. coli* and *Salmonella* spp. were reported at 45.6% and 23%, respectively, in Addis Ababa (16); *Klebsiella* spp. at 29.2% in Gondar (17); and *Salmonella* spp. (19.4%) and *Shigella* spp. (20.2%) in Hawassa (18).

However, our findings were higher than those reported in Nepal, where the isolation rates for *E. coli* and *Klebsiella* spp. were 34.7% and 13%, respectively (19), and also higher than in Nigeria, where *E.coli, Salmonella* spp., *Klebsiella pneumoniae*, and *Proteus mirabilis* were isolated at 24.0%, 13.5%, 19.6%, and 5.01%, respectively (20). The rates also differed from studies in Mekelle, Ethiopia, where *E. coli, Klebsiella* spp., and *Salmonella* spp. were isolated at 13.1%, 16.7%, and 10.7%, respectively (21). These variations may be due to differences in sample size, study period, types of pathogens circulating at the time, effectiveness of infection prevention and control measures, prevalence of enteric pathogens, and the level of disinfection or waste management practices at the hospitals involved.

In our study, most isolates were sensitive to Meropenem (90.4%), which is consistent with findings from Addis Ababa, where 91.8% of isolates were Meropenem-sensitive (22). However, our results differ from those in China, where 51.6% of Enterobacteriaceae isolates were resistant to Meropenem (13).

The Enterobacteriaceae isolates in this study demonstrated high resistance to Amoxicillin-Clavulanic acid (88.4%), Ampicillin (87.0%), Tetracycline (76.5%), and Trimethoprim-sulfamethoxazole (71.9%). Our finding regarding Amoxicillin-Clavulanic acid aligns with a study in Kenya reporting 84.2% resistance (15), and our Ampicillin resistance rate is comparable with the 82.5% reported in Arba Minch, Ethiopia (23). Similarly, a study in Bangladesh reported 75% resistance to Amoxicillin-Clavulanic acid and 63.5% to Ampicillin (14).

A study in China showed high resistance to Tetracycline (74.2%), Trimethoprim-sulfamethoxazole (77.4%), Meropenem (51.6%), Gentamicin (51.6%), and Chloramphenicol (48.4%) (13). These findings are consistent with our resistance data for Tetracycline (76.5%) and Trimethoprim-sulfamethoxazole (71.9%), but differ with regard to Chloramphenicol (20.2%), Gentamicin (24.4%), and Meropenem (9.6%).

In this study, 92.3% of *Salmonella* spp., 87.1% of *E. coli*, and 81.3% of *Proteus* spp. showed resistance to Ampicillin, which is in agreement with findings from Bangladesh, where all *Salmonella* spp. and 75% of both *E. coli* and *P. vulgaris* were resistant to Ampicillin (14).

Our results showed that 75% and 87.5% of Shigella spp. were resistant to Trimethoprim-sulfamethoxazole and Ciprofloxacin, respectively. This contrasts with findings from Nepal, where all *Shigella* spp. were sensitive to both antibiotics (19). The resistance rates of *Klebsiella* spp. to Ciprofloxacin and Tetracycline in our study were 81.5% each, significantly higher than rates reported from Bangladesh, where resistance to these antibiotics was 40% and 50%, respectively (24).

The distribution of antibiotic-resistant bacteria can vary widely between hospitals and regions due to differences in antibiotic use, patient demographics, environmental conditions, and sanitation practices. It is plausible that the isolates in this study were continuously exposed to residual antibiotics in hospital wastewater, increasing the likelihood of harboring multidrug-resistant (MDR) genes that can be transferred to other bacteria.

The current study found that 88.6% of bacterial isolates were multidrug-resistant. This is consistent with previous reports: 85.5% in China (13), 94.8% in Ibadan, Nigeria (25), 81.5% in Northwestern Ethiopia (17), 76.5% in Mekelle (21), 64% in Addis Ababa (22), and 70.4% in Arba Minch (23). In our study, 100% of *Shigella* spp., 93.5% of *E. coli*, 92.8% of *Salmonella* spp., 88.9% of *Klebsiella* spp., and 87.5% of *Proteus* spp. were found to be MDR. Similarly, a study in China reported MDR in 91.4% of *E. coli* and 94.1% of *K. pneumoniae* isolates (13).

The isolation of highly MDR pathogens from hospital wastewater indicates that releasing untreated wastewater into nearby rivers may have serious ecological and public health consequences. It can adversely affect aquatic life, animals, plants, and humans, and may facilitate the horizontal transfer of resistance genes to other bacterial populations. Therefore, it is essential that hospital wastewater is treated before being released into the environment.
